# 2717. Beware the bacteria: risk of bacterial infections persists more than 60 days post CAR-T

**DOI:** 10.1093/ofid/ofad500.2328

**Published:** 2023-11-27

**Authors:** Poonam Mathur, David J Riedel, Jacqueline T Bork, Katya Prakash, Nancy Hardy, John W Baddley

**Affiliations:** University of Maryland, Baltimore, Maryland; Institute of Human Virology, University of Maryland School of Medicine, Baltimore, MD; University of Maryland School of Medicine, Severna Park, Maryland; University of Maryland School of Medicine, Severna Park, Maryland; University of Maryland School of Medicine, Severna Park, Maryland; University of Maryland School of Medicine, Severna Park, Maryland

## Abstract

**Background:**

Immunosuppressive conditioning regimens for chimeric antigen receptor-modified T cells (CAR-T) and subsequent T cell dysfunction increase risk for infection. Prior studies of infection risk after CAR-T have found bacterial infections are most common in the first month, and viral infections are more frequently seen after 30 days. We evaluated longer-term infection outcomes, up to 180 days post CAR-T infusion.

**Methods:**

We performed a retrospective chart review of 144 patients who received CAR-T infusion at our institution between March 2018 and March 2022. We collected data on demographics, type and timing of infections, onset and treatment of Immune Effector Cell-Associated Neurotoxicity Syndrome (ICANS) or Cytokine Release Syndrome (CRS), and mortality up to 180 days after CAR-T infusion. We determined if ICANS, CRS, or treatment for either was associated with infections using Chi-square test.

**Results:**

The mean age of patients in our cohort was 59 years. Most patients were male (65%), White (66%), and had not received a prior stem cell transplant (78%). Fifty-one (35%) patients had at least one infection during the study period (29% bacterial, 13% viral, 2% fungal). Mortality was 23% at 180 days. Most infections occurred within 60 days post CAR-T (Figure 1). Infections occurring between days 61 and 180 were mostly bacterial. In addition, ICANS ≥grade 3 and CRS ≥grade 2 occurred in 22 (15%) and 54 (38%) patients, respectively; both were significantly associated with bacterial infection (p< 0.05).

Figure 1

**Figure:**
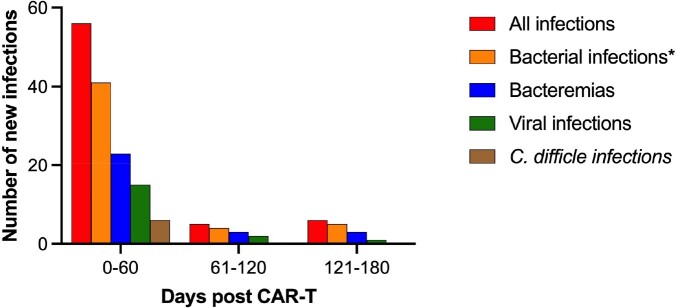

Infections 180 days after CAR-T. *includes bacteremia, pneumonia, and skin and soft tissue infections.

**Conclusion:**

This cohort analyzed long-term infection outcomes post CAR-T. Most infections occurred early after CAR-T, within 60 days, and ICANS and CRS were associated with bacterial infection. We found that infections still persisted late after CAR-T, between Days 61 and 180, and were predominately bacterial, rather than viral or fungal. The low rates of fungal infection throughout the study period may be due to our institution’s fungal prophylaxis protocol. Future analyses will elucidate risk factors for infections in the late CAR-T period.

**Disclosures:**

**David J. Riedel, MD, MPH** , Gilead: Advisor/Consultant

